# Carboxylato-pillar[6]arene-based fluorescent indicator displacement assays for the recognition of monoamine neurotransmitters†

**DOI:** 10.1039/c9ra03241j

**Published:** 2019-05-29

**Authors:** Adrien Paudics, Miklós Kubinyi, István Bitter, Márton Bojtár

**Affiliations:** Department of Physical Chemistry and Materials Science, Budapest University of Technology and Economics 1521 Budapest Hungary; Institute of Environmental and Materials Chemistry, Research Centre for Natural Sciences, Hungarian Academy of Sciences 1519 Budapest P.O. B. 286 Hungary; Department of Organic Chemistry and Technology, Budapest University of Technology and Economics 1521 Budapest Hungary bojtar.marton@ttk.mta.hu; Chemical Biology Research Group, Institute of Organic Chemistry, Research Centre for Natural Sciences, Hungarian Academy of Sciences 1519 Budapest P.O. B. 286 Hungary

## Abstract

The complexation of three cationic fluorescent dye guests with the anionic host carboxylato-pillar[6]arene (WP6) was investigated by optical and NMR spectroscopy. Among the selected indicators – a stilbazolium dye (i1) and two naphthalimide derivatives with positively charged ‘anchor’ groups (i2 and i3) – i1 gave a large turn-on, i2 and i3 a large turn-off fluorescence response to the complexation. The size selectivity of the complex formation of pillararenes was demonstrated by comparing the binding constants of the complexes of the three indicators with WP6 and its smaller homologue, WP5. The systems WP6·i1 and WP6·i2 were tested as indicator displacement assays for the sensing of monoamine neurotransmitters. The WP6·i1 system functioned as a turn-off, the WP6·i2 system as a turn-on sensor for neurotransmitters, and both assays showed a good selectivity to histamine over the other neurotransmitter analytes.

## Abstract 2

The complexation of three cationic fluorescent dye guests with the anionic host carboxylato-pillar[6]arene (WP6) was investigated by optical and NMR spectroscopy.

## Graphical abstract

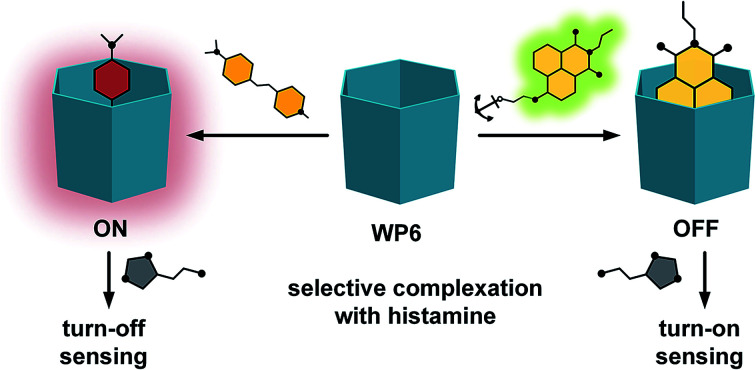

## Introduction

Since the emergence of supramolecular chemistry, the recognition of molecules with biological interest is a topic of great importance.^1–3^ Artificial receptor molecules operating through host–guest interactions capable of selective molecular recognition can be applied in supramolecular analytical systems to detect and quantify the analytes of interest.^4–6^ Macrocycles, due to their inherent structural nature are ideal hosts for various biomolecules, however, attaching a signaling group to the water soluble derivatives is highly challenging.^7^ This can be overcome by using fluorescent indicator displacement (FID) assays, exploiting the competitive binding of a fluorescent indicator to the macrocycle.^8,9^

The hosts in most of the FID-based sensing systems reported so far were cyclodextrins,^10,11^ calixarenes^12–15^ or cucurbiturils,^16–19^ however, with the appearance of pillararenes in 2008,^20^ an interesting new possibility emerged for expanding the toolbox of FID-type supramolecular assays. Soon these macrocycles became the new key players of supramolecular chemistry due to their easy synthesis, versatile functionalization and diverse host–guest properties.^21–25^ In particular, water soluble pillararenes are prosperous hosts for various guests of biological importance.^26–28^ Carboxylato-pillar[5]arene forms complexes with viologens (paraquat),^29,30^ various amines and basic amino acids^31^ that can be exploited to compile FID assays for these analytes.^32–34^ Cationic, ammonium-containing pillar[5]arene was used as receptor in a FID sensor for various phenols.^35^ Amongst the higher homologues, carboxylato-pillar[6]arene can also recognise paraquat,^36^ furthermore, ammonium-pillar[6]arene forms an exceptionally strong and selective complex with ATP^37^ that can be used in nucleotide recognition.^38^ Recently, the complexation of carboxylato-pillar[6]arene with choline was also described.^39^ The widespread success of water soluble pillararenes encouraged us to extend the current set of supramolecular systems based on these hosts for further applications.

**Fig. 1:**
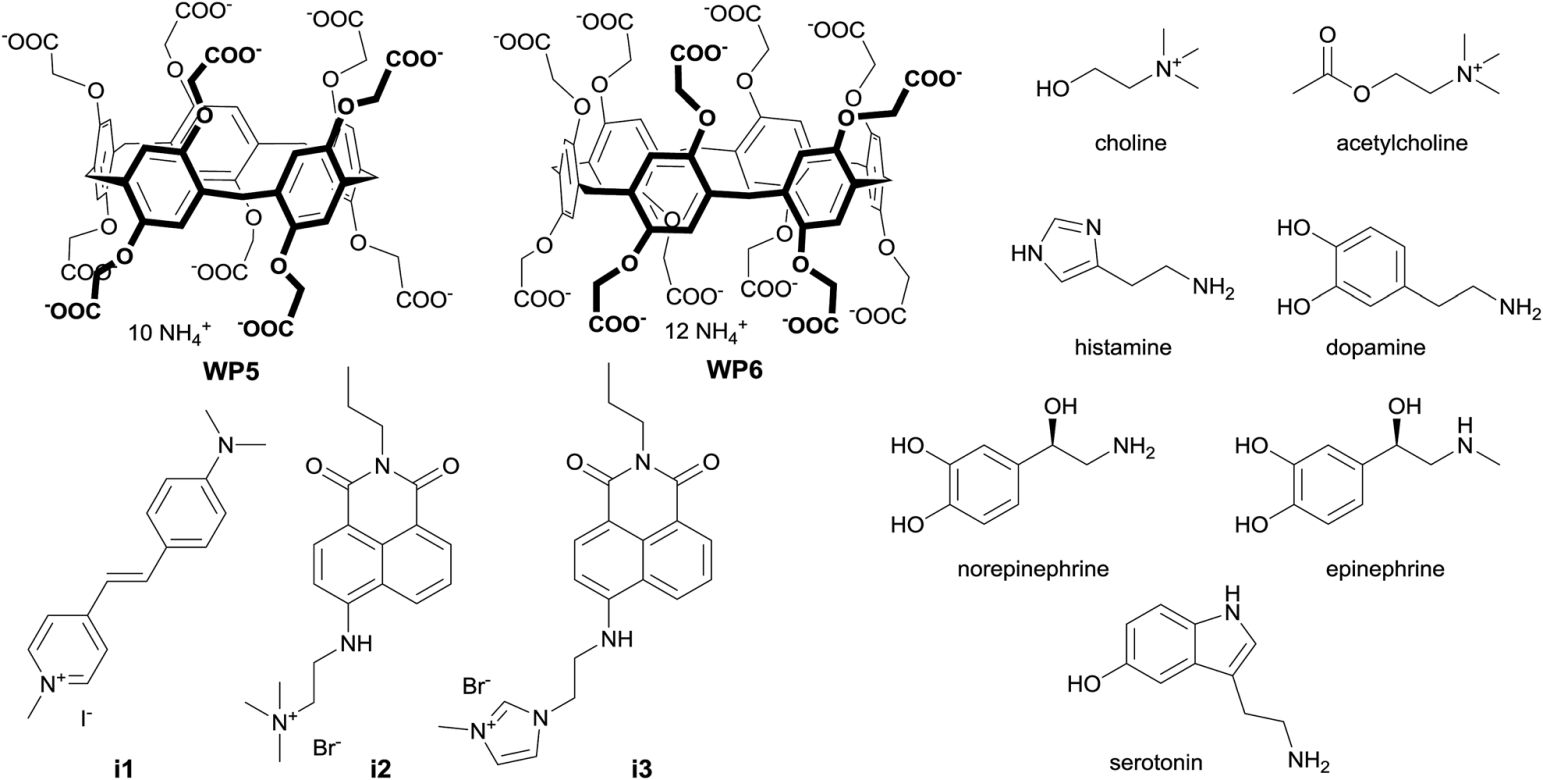
Structure of the macrocycles, indicators and analytes.

In our previous work, we described the complexation of some stilbazolium dyes with WP5.^33^ In the case of i1 (4-dimethylaminostyryl-*N*-methyl-pyridinium iodide, DAST) an intense turn-on fluorescence and a colourimetric response was observed upon complexation. We have also investigated naphthalimide derivatives (i2 and i3) possessing an anchor moiety (trimethylammonium and imidazolium, respectively) to promote binding to the negatively charged WP5.^34^ In this case, turn-off fluorescence followed the partial inclusion of the indicators. Therefore, we turned our attention towards these results to evaluate the complexation of these dyes with the 6-membered homologue, WP6. Due to its larger cavity, WP6 can efficiently bind larger cationic guests than the smaller WP5 which prompted us to evaluate these FID assays with various monoamine neurotransmitters (see Fig. 1 for structures).

Since the recognition and quantification of these analytes is highly important for both neuroscience and diagnostics, numerous solutions exist for their detection.^40–42^ Most of these sensing systems are based on chromatographic or electrochemical methods, or utilise tailored nanodevices. The fluorescent probes for monoamine derivatives are usually chemodosimeters reacting irreversibly with the analyte,^43–47^ however, this binding pattern generally lacks selectivity. For the recognition of trimethylammonium-containing acetylcholine, sulfonato-calixarenes were widely used as a host molecules.^12,13,48^

**Fig. 2:**
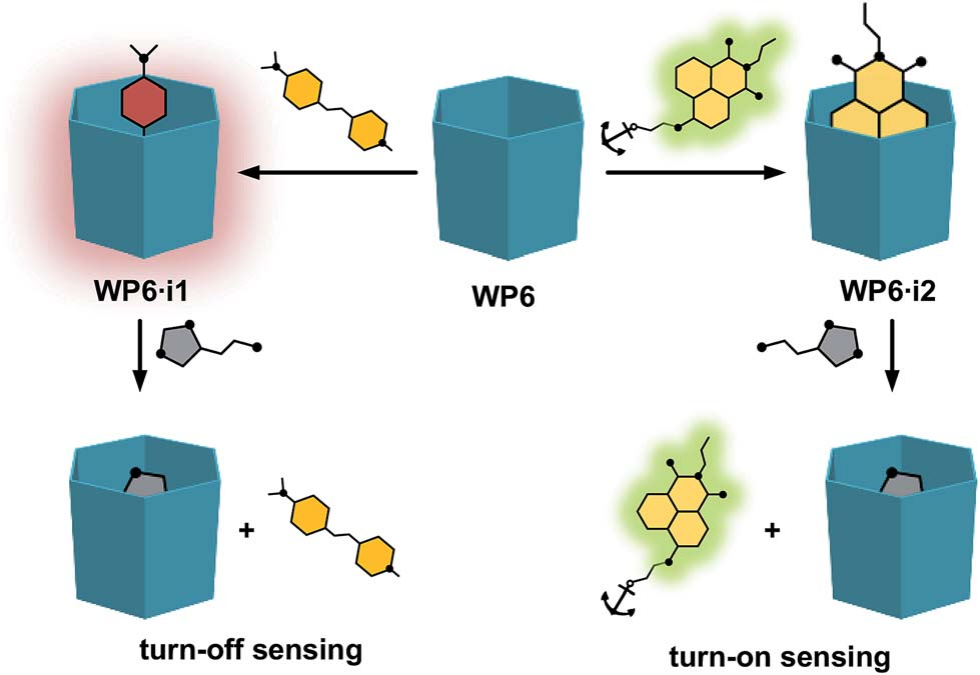
Schematic representation of the indicator displacement assays WP6·i1 and WP6·i2 for histamine.

As will be shown, both the systems WP6·i1 and WP6·i2 worked as FID assays for neurotransmitters, showing selectivity towards histamine over the other analytes (Fig. 2). In addition, comparing the properties of the complexes of the indicators with WP5, the size selective complexation of pillararenes was also demonstrated.

## Results and discussion

### Complexation of fluorescent indicators with WP6

**Fig. 3:**
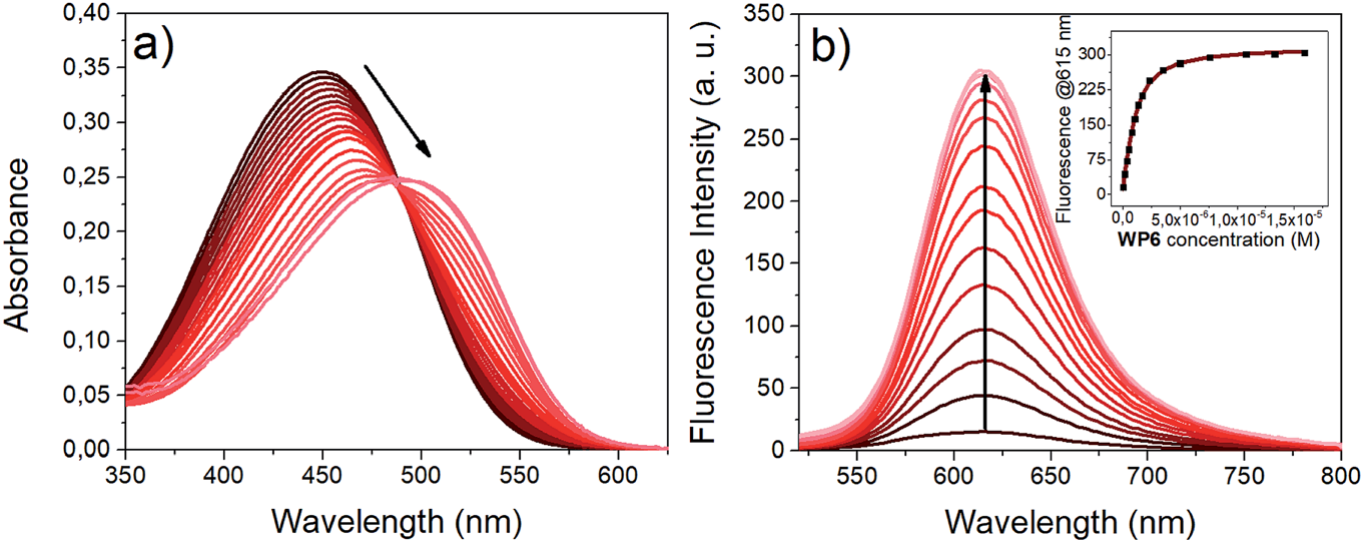
Complexation of i1 with WP6 in HEPES buffer (pH 7.4). (a) Absorption spectra (10.0 μM i1, 0 to 1.74 equiv. WP6) (b) fluorescence spectra (1.0 μM i1, 0 to 16 equiv. WP6, *λ*_ex_ (isosbestic point): 482 nm). The inset shows the fluorescence emission at 615 nm *vs.*WP6 concentration (black dots: experimental data, red curve: result of non-linear fitting).

Upon addition of WP6 to the HEPES buffered solution of i1, remarkable changes were observed both in the absorption and emission spectra (Fig. 3). The complexation caused a significant redshift in the absorption maximum and an intense fluorescence enhancement. These changes can be explained in terms of the existence of an emissive directly excited charge transfer (CT) state and a dark twisted intramolecular charge transfer (TICT) state on the S_1_ potential energy surface of i1. The dipole moment is significantly higher in the S_1_(CT) state than in the ground state, therefore the S_1_(CT) state molecule is stabilised in the environment of the high negative charge density of the WP6 host, leading to the redshift of the absorption band. In the dark TICT state, presumably the phenyl or the pyridinium group is rotated around the bonds connecting them to the central double bond.^49^

This twisting motion is restricted for the encapsulated i1 guest, resulting in fluorescence enhancement.

**Fig. 4:**
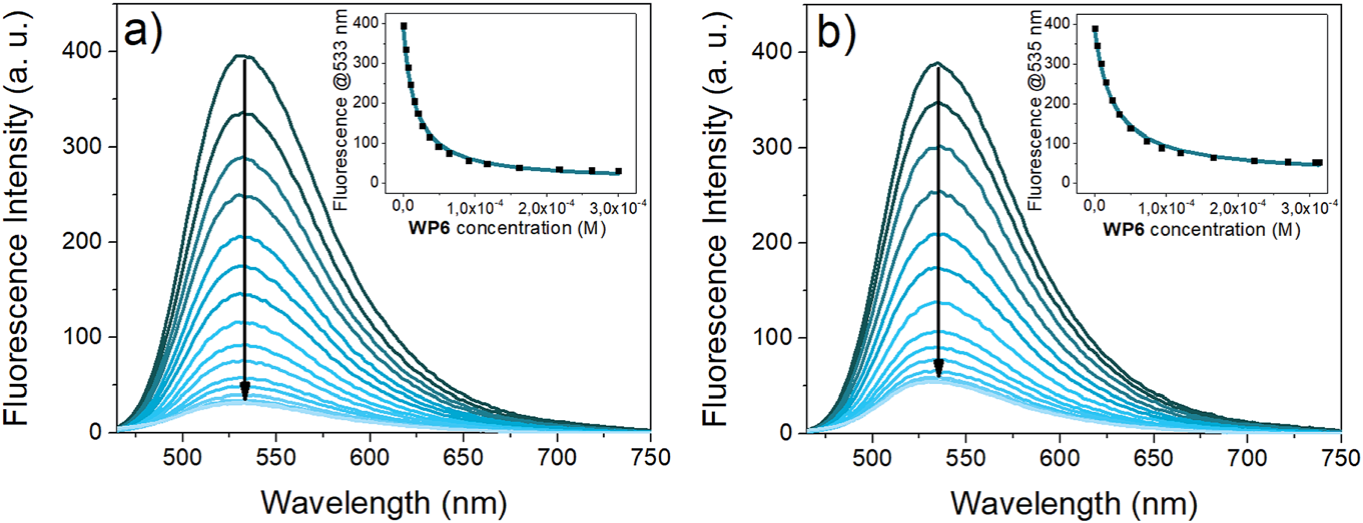
Fluorescence titration of the guests (a) i2 (1 μM, *λ*_ex_: 433 nm) (b) i3 (1 μM, *λ*_ex_: 438 nm) with WP6 (0 to 300 equivalents) in 0.02 M HEPES buffer (pH 7.4). The insets show the fluorescence emission at 533 nm *vs.*WP6 concentration (black dots: experimental data, cyan curve: result of non-linear fitting).

The absorption spectra of the naphthalimide indicators i2 and i3 remained almost unaffected by the addition of WP6 (Fig. S1 and S2 in the ESI†). In contrast, their fluorescence was strongly quenched by WP6 (see Fig. 4), at large WP6 excesses the intensities of both i2 and i3 underwent to an about 10-fold decrease.

The fluorescence of 4-amino-naphthalimide indicators can be controlled *via* a photoinduced electron transfer (PET) mechanism.^49^ 4-Amino-naphthalimides are weakly fluorescent due to PET from the amino to the naphthalimide group.

Attaching a positively charged ammonium group *via* an ethylene spacer to the 4-amino group – like the tetramethylammonium in i2 or the imidazolium in i3 – the PET is suppressed, the fluorescence becomes stronger.^50^ Upon complexation by WP6, the proximity of the negative charges on the carboxylato groups shields the positively charged anchor groups, the PET in the indicators is recovered, leading to a substantial reduction of fluorescence intensity.

The association constants (*K*_1_) for the WP6 indicator complexes were determined by a non-linear fitting to the fluorescence spectra obtained in the titration experiments.^51,52^ The 1 : 1 stoichiometry of the complexes was checked by the molar ratio method, using the absorbance data of WP6-i1 mixtures and NMR shifts of WP6-i2 mixtures (see Fig. S2 and S3 in the ESI,† the poor solubility of WP6·i1 did not afford NMR measurements). The binding constants, together with the spectral data of the complexes are collected in Table 1. For comparison, the *K*_1_ values and spectral data of the WP5 complexes of the three indicators, reported in our earlier studies, are also shown in the table.

**Table 1:** Association constants and spectral data of the complexes of indicators i1, i2 and i3 with WP5 and WP6. For comparison, the spectral data of the uncomplexed indicators are also shown

	log *K*_1_ (*K*_1_ in M^−1^)	*λ*_abs_ (nm)	*ε* (M^−1^ cm^−1^)	*λ*_em_ (nm)	*Φ*
i1		449	29 200	615	0.002
WP5·i1^a^	6.11	515	14 300	607	0.041
WP6·i1	6.26	501	15 900	615	0.035
i2			14 900	533	0.32
WP5·i2^b^	3.68	433	14 800		0.06
WP6·i2	4.81	428	15 900		0.03
i3		438	18 800	535	0.20
WP5·i3^b^	5.45		14 800		0.02
WP6·i3	4.61		14 700		0.02

a From ref. 33.

b From ref. 34.

The similarly high values of the stability constants for WP5·i1 and WP6·i1 demonstrates that they are both strong complexes and that the complexation of this indicator does not depend on the ring size of the host. In contrast, the inclusion of the anchored naphthalimides exhibits a pronounced size-selectivity. The *K*_1_ value for the WP6·i2 complex is more than an order of magnitude higher than the value of *K*_1_ for WP5·i2, *i.e.* the bulky tetraalkylammonium anchor fits better into the larger cavity of six-membered macrocyclic host.

Conversely, the indicator with the smaller imidazolium anchor, i3 forms a more stable complex with the macrocycle of tighter cavity, WP5.

**Fig. 5:**
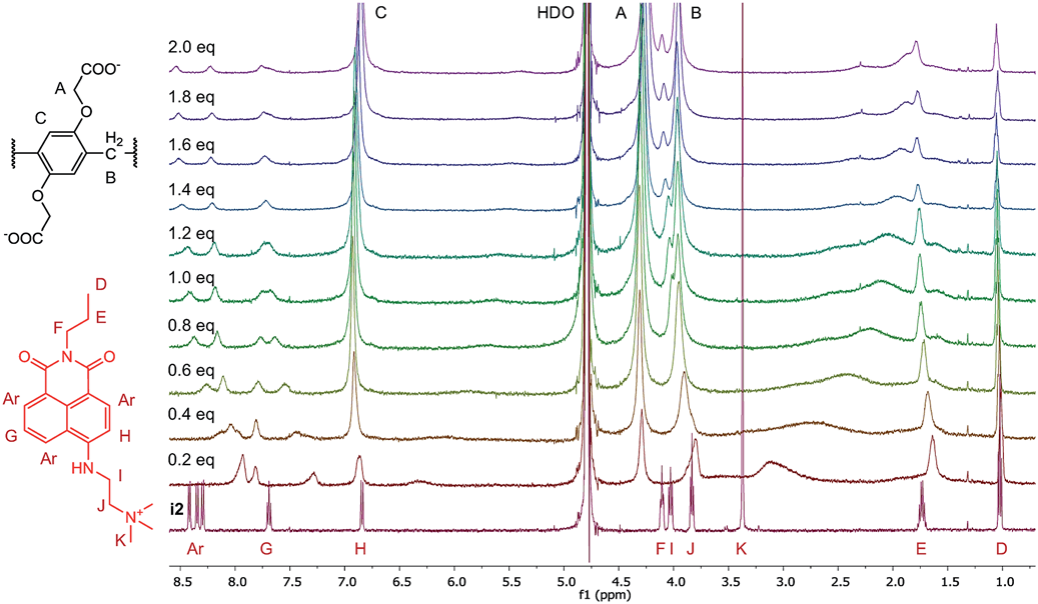
^1^H-NMR spectra (500 MHz, D_2_O, 0.5 mM) of i2 in the presence of 0 to 2.0 equivalents of WP6.

The structures of the indicator–macrocycle complexes were further evaluated using ^1^H-NMR spectroscopy. Unfortunately, the solubility of the WP6·i1 complex was low, not affording a spectrum with a reasonably good S/N ratio. Valuable information could be obtained, however, from the spectrum of WP6·i2 (Fig. 5).

A large alteration in the complex spectrum can be observed which is attributable to the inclusion of i2 in the cavity of WP6. Both the anchor moiety (protons I, J and K) and the aromatic protons are significantly shifted that indicates not only partial inclusion as in the case of WP5 but complete threading of the guest molecule. This is a large difference from the structure of the complex WP5·i2, in which only the anchor moiety intrudes into the pillararene cavity, providing an interesting example for the size-selective complexation by pillararene supramolecular receptors.

### Fluorescent indicator displacement

**Fig. 6:**
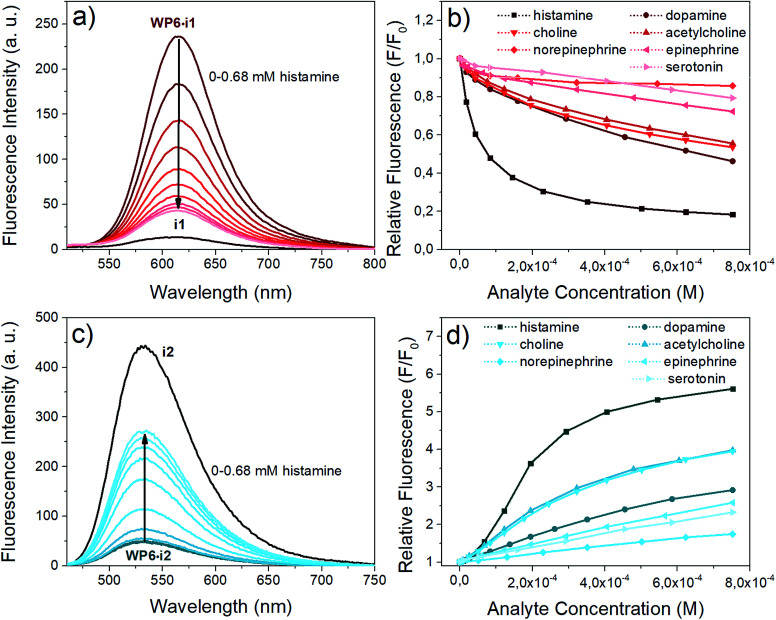
Indicator displacement of the complexes with various neurotransmitter analytes in HEPES buffer (pH 7.4). (a) Fluorescence spectra of WP6·i1 (0.9 μM i1, 5.8 μM WP6, *λ*_ex_: 482 nm) upon addition of histamine (0 to 0.68 mM) (b) relative fluorescence values of WP6·i1 at 615 nm upon addition of different analytes (c) fluorescence spectra of WP6·i2 (0.9 μM i1, 126 μM WP6, *λ*_ex_: 438 nm) upon addition of histamine (0 to 0.68 mM) (d) relative fluorescence values of WP6·i2 at 533 nm upon addition of different analytes.

Next, we aimed at using two of our indicator–macrocycle complexes for chemosensing purpose, namely, WP6·i1 and WP6·i2. Not only their association constants are different but opposite fluorescence responses to the addition of analytes could be expected from these two supramolecular analytical systems. This dual reactivity might be a useful feature for differential sensing and molecular logic gates.^53^ To achieve large fluorescence responses, we selected the macrocycle concentrations to reach exactly 90% complexation degree for both systems. At this concentration, 6 and 140 equivalents of WP6 were required for i1 and i2, respectively. The changes of the fluorescence spectra of the two assays in the presence of the neurotransmitters are illustrated in Fig. 6.

The opposite fluorescence responses are demonstrated on the spectra obtained with histamine as analyte – this neurotransmitter induces the largest changes (see Fig. 6a and c). The addition of histamine to the WP6·i1 assay leads to a large turn-off fluorescence response, even at a low analyte concentration. In the case of WP6·i2, a significant, almost 6-fold fluorescence enhancement was reached at higher histamine concentrations. The selective complexation of histamine with WP6 is apparent when comparing the fluorescence responses of the different analytes (Fig. 6b and d).

At low concentrations of histamine, the WP6·i1 system is more sensitive, the detection limit is ≈ 2 × 10^−7^ M. The sensitivity of the WP6·i2 system in the initial range is lower, due to the high relative excess of the WP6 host. Both systems respond to choline and acetylcholine, but to a much weaker extent. The two assays do not distinguish these two analytes which can be rationalised by their structural similarity and identical (trimethylammonium group) recognition site. The other monoamine analytes applied (dopamine, norepinephrine, epinephrine, serotonin) induced even weaker spectral changes.

The stability constant of the histamine–WP6 complex, *K*_2_ was calculated from the fluorescence spectra of the i2-WP6–histamine and i1-WP6–histamine ternary systems. A non-linear fitting was carried out to the fluorescence intensities at the band maxima, using the algorithm developed by Anslyn *et al.*^54^*K*_2_ was considered a single fitting parameter, with a common value for the two sets of spectral data, the values of the *K*_1_ stability constants for the WP6·i1 and WP6·i2 complexes in Table 1, obtained from the spectra of the pillararene-indicator mixtures were retained. This calculation yielded a value of *K*_2_ = 2.1 × 10^5^ M^−1^ for the WP6–histamine complex.

As can be seen in Fig. S5 and S6 in the ESI,† the calculations reproduced the experimental data successfully – in case of the WP6-i1–histamine systems the agreement was very good – demonstrating the validity of this simple reaction scheme for both the turn-off assay WP6·i1 and the turn-on assay WP6·i2.

**Fig. 7:**
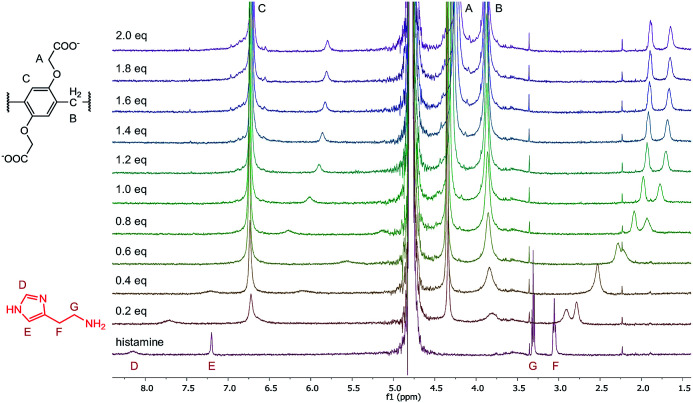
^1^H-NMR spectra (500 MHz, D_2_O, 0.5 mM) of histamine in the presence of 0 to 2.0 equivalents of WP6.

To further evaluate the complexation of the analytes, ^1^H-NMR experiments were performed in the case of histamine, dopamine, choline and acetylcholine. The ^1^H spectra of the titration of histamine with WP6 are shown in Fig. 7. As can be seen in the figure, the addition of WP6 resulted in the upfield shift and broadening of all the four proton signals of histamine, indicating the inclusion of the whole molecule. The significant upfield shift is the result of the inclusion in the aromatic rings of the macrocycle. The signals in the NMR spectra of dopamine, choline and acetylcholine changed in a similar manner upon the addition of WP6 (Fig. S7–S9†) with the exception of the signal of the acetyl group of acetylcholine. In that case, only a small shift was detected that might be due to the exclusion of the acetyl group from the cavity of the macrocycle which explains why the WP6-based assays did not differentiate between choline and acetylcholine. The elongation of the cavity (*i.e.* synthesising macrocycles built up from ‘longer’ monomer molecules) might result in the strongly desired selective recognition of acetylcholine.^55^

**Fig. 8:**
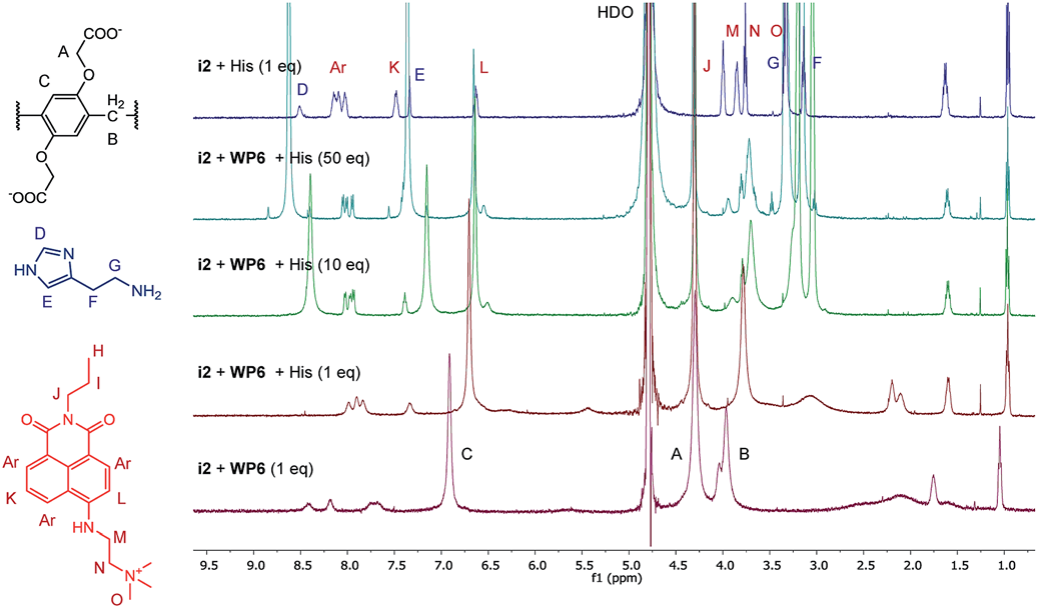
^1^H-NMR spectra (500 MHz, D_2_O, 0.5 mM) of the indicator displacement of i2-WP6 mixture in the presence of histamine in different concentrations. The uppermost spectrum is a mixture of i2 and histamine.

The changes in the NMR spectrum of the assay WP6·i2 in course of the indicator displacement by histamine are illustrated in Fig. 8. The spectrum of i2 with 1 equivalent histamine, in the absence of WP6 (uppermost spectrum) demonstrates the lack of interaction between these two guest molecules. Starting from the WP6-i2 1 : 1 binary mixture (lowermost spectrum), the displacement of the indicator is clearly observable even in the spectrum of the 1 : 1 : 1 ternary mixture. In this spectrum, the broadening and the shifting of the signals of i2 become less pronounced, the signals are closer to the original ones of i2. Accordingly, the positions and shapes of the signals of histamine are changed less in the spectrum of the 1 : 1 : 1 ternary system than in the spectrum of the WP6–histamine binary mixture (Fig. 6). This is a clear evidence that i2 and histamine bind to WP6 in commensurable ratios, in accordance with the close values of the stability constants of the WP6·i2 and WP6·histamine complexes. Adding histamine to the assay in higher excesses, the spectral features of i2 are gradually recovered.

## Experimental

The indicators i1, i2 and i3 were synthesised as described in our earlier papers.^33,34^ The synthesis of WP6 (ammonium salt) was carried out as reported^56–58^ with slight modifications in the procedure and described in the ESI† in detail. The starting materials and solvents were purchased from commercial suppliers and used without further purification. The neurotransmitters were obtained from Sigma (choline and acetylcholine as chloride salts, histamine as dihydrochloride, serotonin and dopamine as hydrochloride).

The absorption and fluorescence spectroscopic experiments were carried out in HEPES buffers of pH 7.4. The fluorescence quantum yields were determined using Coumarin 153 as reference. The absorption spectra were recorded on an Agilent 8453 spectrometer. The fluorescence spectra were measured on a PerkinElmer 50B spectrofluorimeter. The NMR spectra were acquired on a 500 MHz Bruker Avance DRX-500 spectrometer. All the spectral measurements were carried out at 25 °C.

## Conclusion

We studied the inclusion complexes of three fluorescent indicators – a stilbazolium dye and two ‘anchored’ naphthalimide derivatives – with carboxylato-pillar[6]arene, WP6. The stilbazolium dye (i1) gave turn-on, the naphthalimides (i2 and i3) turn-off fluorescence responses upon complexation. The complexes of the three indicators with WP6 and its five-membered ring homologue, WP5 were compared and in the case of the two naphthalimide derivatives an interesting size selectivity was found. Furthermore, two of the WP6-indicator supramolecular systems were selected as fluorescent indicator displacement assays for the detection of neurotransmitters. The two systems showed opposite fluorescence responses and good selectivity towards histamine. We believe that this work can further extend the application of this versatile macrocycle family, especially their still unexplored supramolecular analytical potential.

## Conflicts of interest

There are no conflicts to declare.

## Supplementary Material

RA-009-C9RA03241J-s001
