# Supplementary material for: Carboxylato-pillar[6]arene-based fluorescent indicator displacement assays for the recognition of monoamine neurotransmitters
Source: RSC Adv. 2019 May 29;9(29):16856–62. doi: 10.1039/c9ra03241j (PMC9064442; doi:10.1039/c9ra03241j)
Supplement: RA-009-C9RA03241J-s001 [file RA-009-C9RA03241J-s001.pdf]

Electronic Supplementary Material (ESI) for RSC Advances.
This journal is © The Royal Society of Chemistry 2019

Electronic Supplementary Information (ESI)

Carboxylato-pillar[6]arene-based fluorescent indicator displacement assays for the recognition of monoamine neurotransmitters

Adrien Paudics[a], Miklós Kubinyi[a,b], István Bitter[c] and Márton Bojtár*[c,d]

[a] Department of Physical Chemistry and Materials Science, Budapest University of Technology and Economics, 1521 Budapest, Hungary

[b] Institute of Materials and Environmental Chemistry, Research Centre for Natural Sciences, Hungarian Academy of Sciences, 1519 Budapest, P. O. B. 286, Hungary

[c] Department of Organic Chemistry and Technology, Budapest University of Technology and Economics, 1521 Budapest, Hungary

[d] Chemical Biology Research Group, Institute of Organic Chemistry, Research Centre for Natural Sciences, Hungarian Academy of Sciences, 1519 Budapest, P. O. B. 286, Hungary

*corresponding author, bojtar.marton@ttk.mta.hu

1. *Synthesis of **WP6***
2. *Absorption spectra of **i2** and **i3** with **WP6***
3. *Stoichiometry determination*
4. *Non-linear fitting results of the indicator displacement*
5. *NMR spectra of **WP6** with choline, acetylcholine and dopamine*

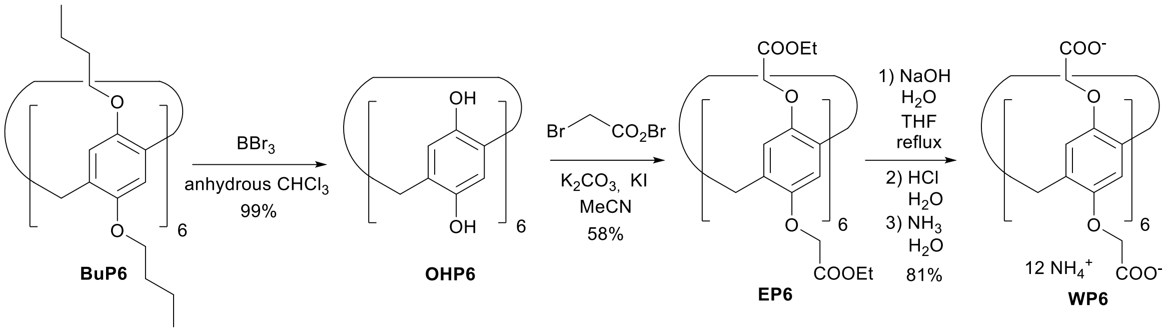

Scheme S1. Synthesis of **WP6**. Note that the synthesis is based on other literature reports (references in the main text: 56-58)

*Dodecabutyl-pillar[6]arene (**BuP6**)*

The macrocyclization reaction was performed using choline chloride/$FeCl_3$ catalyst according to ref. 56 in the main text.

*Dodecahydroxy-pillar[6]arene (**OHP6**)*

To a dried round-bottom flask was added 1.88 g (1.35 mmol) **BuP6** dissolved in 50 ml anhydrous chloroform. The solution was cooled on ice and 4 ml (10.4 g, 41.5 mmol, 31 eq.) boron tribromide was added slowly. The mixture was stirred for 72 hours at room temperature. After completition, 100 ml water was added with cooling and constant Ar flow. The mixture was stirred for 1 hour and filtered. The white precipitate was washed with 1 M hydrochloric acid, water and chloroform multiple times and dried with the exclusion of oxygen. It is advised to use all of the product since it is unstable and readily oxidized in a couple of hours when exposed to air. Yield: 1.00 g (99%) white powder. ^{1}H-NMR (300 MHz, acetone-d_6) δ 7.70 (s, 10H, OH), 6.54 (s, 10H, ArH), 3.67 (s, 10H, CH_2) ^{13}C-NMR (75 MHz, acetone-d_6) δ 148.2; 127.1; 118.1; 30.3

*Dodecaethoxycarbonylmethyl-pillar[6]arene (**EP6**)*

The freshly prepared **OHP6** was suspended in 100 ml acetonitrile and deoxygenated with constant Ar bubbling for 20 minutes. Then, 8.4 g (61 mmol, 45 eq.) K_2CO_3 and 3.32 ml (4.98 g, 30.0 mmol, 22 eq.) bromoacetic acid ethylester was added and the mixture was heated at reflux for 48 hours under Ar atmosphere. It is highly important to exclude oxygen in this reaction. After that, the solvent was evaporated and separated between dichloromethane and water. The aqueous phase was extracted three times with dichloromethane, the collected organic phase was washed with water, dried on $MgSO_4$ and evaporated. The resulting crude product was stirred in 25 ml methanol overnight, filtered, washed with methanol and a small amount of cold acetone and dried. Yield: 1.30 g (58%) white powder.^{1}H-NMR (300 MHz, $CDCl_3$) δ 6.90 (s, 12H, ArH), 4.49 (s, 24H, O-CH_2-CO), 4.21 (q, J = 7,1 Hz, 24H, O-CH_2-CH_3), 3.89 (s, 12H, Ar-CH_2), 1.24 (t, J = 7,1 Hz, 36H, CH_3) ^{13}C-NMR (126 MHz, $CDCl_3$) δ 169.5; 150.2; 128.1; 115.5; 66.2; 60.9; 31.1; 14.3

*Dodecacarboxymethyl-pillar[6]arene dodecaammonium salt (**WP6**)*

To a solution of 1.28 g (0.72 mmol) **EP6** in 40 ml tetrahydrofuran was added 25 ml 15% (w/w) aqueous sodium hydroxyde solution. The mixture was heated at reflux for 20 hours then cooled on ice. The pH of the solution was set to 2-3 with 3 M hydrochloric acid and the resulting precipitate was filtered and washed thoroughly with water. The off-white product was suspended in water and 1 ml of 25% aqueous ammonia solution was added. The insoluble particles were removed by filtration and the water and excess ammonia were evaporated. Yield: 0.95 g (81%) off-white powder. ^{1}H-NMR (300 MHz, D_2O) δ 6.70 (s, 12H, ArH), 4.20 (s, 24H, O-CH_2), 3.89 (s, 12H, Ar-CH_2) ^{13}C-NMR (75 MHz, D_2O) δ 178.9; 152.2; 130.5; 118.0; 70.1; 32.6. ESI-HRMS: calculated for $[M\text{-}11NH_4\text{+}12H]^+$ m/z = 1446.3208 measured: 1446.3218

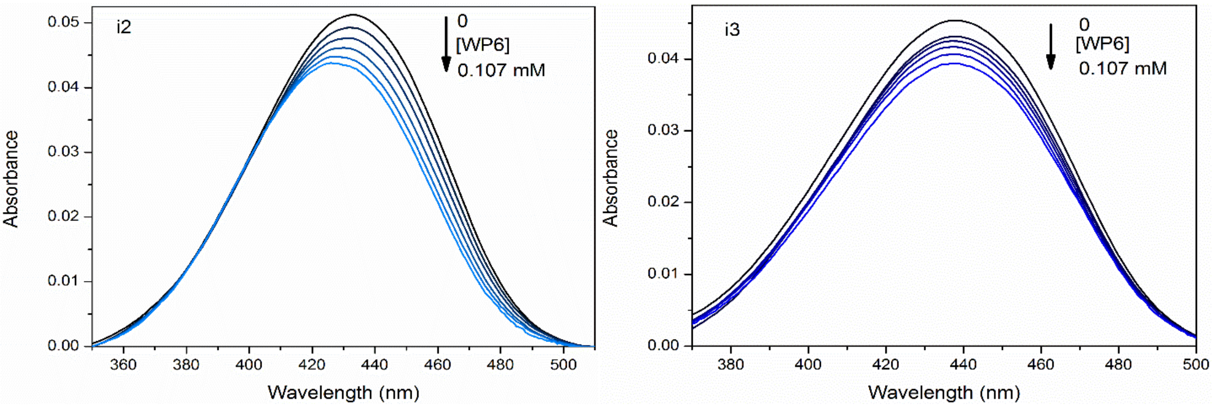

Figure S1. Absorption spectra of 3 μM solutions of the naphthalimide indicators **i2** and **i3** at different concentrations of **WP6**.

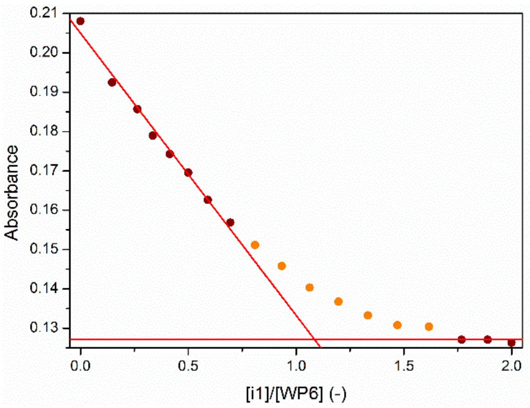

Figure S2. Plot of the absorbances of 7.5 μM solutions of **i1** at 449 nm, containing 0-2 equivalents of **WP6**.

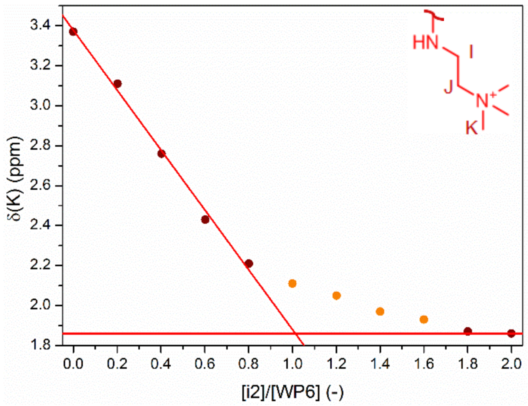

Figure S3. Change of the chemical shift of proton 'K' of **i2** (0.5 mM in D_2O) in the presence of 0-2 equivalents of **WP6**.

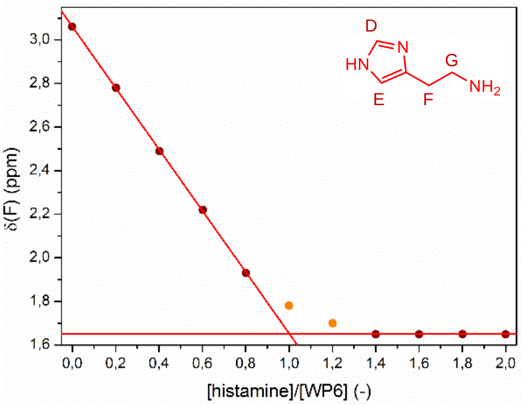

Figure S4. Change of the chemical shift of proton 'F' of histamine (0.5 mM in D_2O) in the presence of 0-2 equivalents of **WP6**.

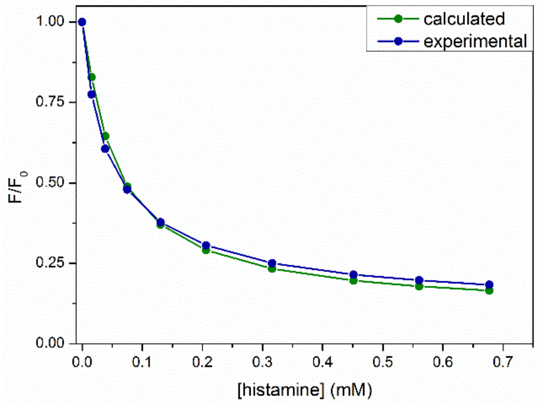

Figure S5. Relative fluorescence intensities of displacement assay **WP6-i1** at λ_{em} = 615 nm ([**i1**]$_0$ = 0.9 μM, ([**WP6**]$_0$ = 5.8 μM, λ_{ex} = 482 nm) at different histamine concentrations. The calculated values were obtained with binding constants K(**WP6·i2**) = $1.86{\cdot}10^5$ $M^{-1}$and K(**WP6·**histamine) = $2.1{\cdot}10^5$ M^{-1}.

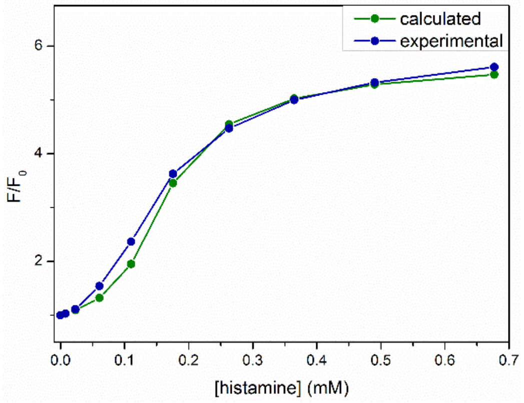

Figure S6. Relative fluorescence intensities of displacement assay **WP6-i2** at λ_{em} = 533 nm ([**i2**]$_0$ = 0.9 μM, ([**WP6**]$_0$ = 140 μM, at λ_{ex} = 438 nm) at different histamine concentrations. The calculated values were obtained with binding constants K(**WP6·i2**) = 1.86 10^5 $M^{-1}$and K(**WP6·**histamine) = 2.1·10^5 M^{-1}.

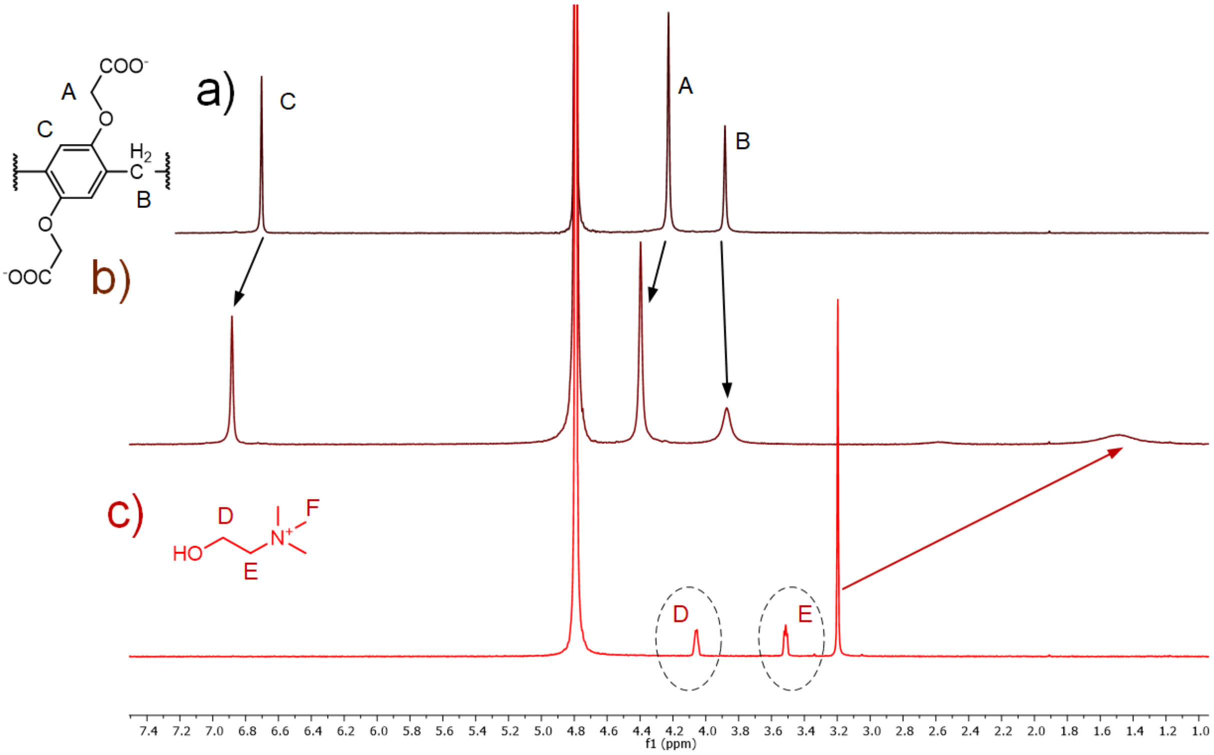

Figure S7. ^{1}H-NMR spectra (500 MHz, D_2O, 1 mM) of a) **WP6** b) **WP6** + choline c) choline. The dashed ovals show the disappearing signals

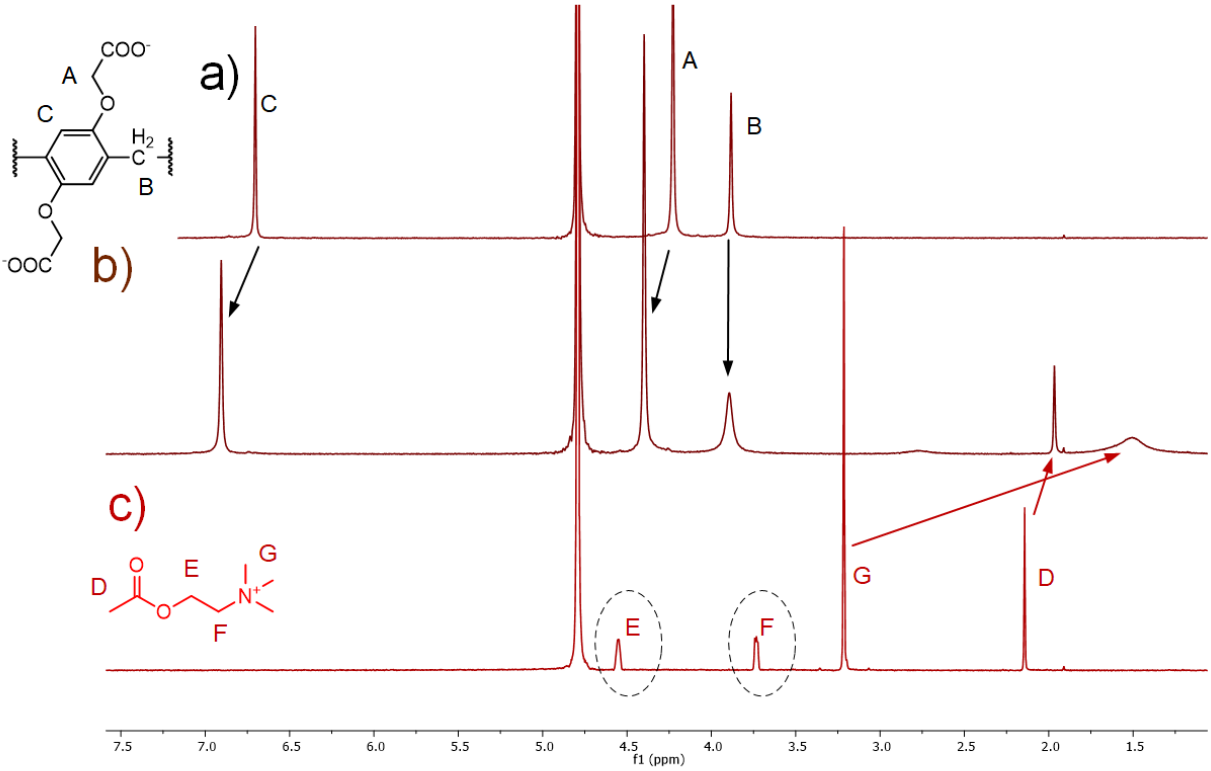

Figure S8. ^{1}H-NMR spectra (500 MHz, D_2O, 1 mM) of a) **WP6** b) **WP6** + acetylcholine c) acetylcholine. The dashed ovals show the disappearing signals

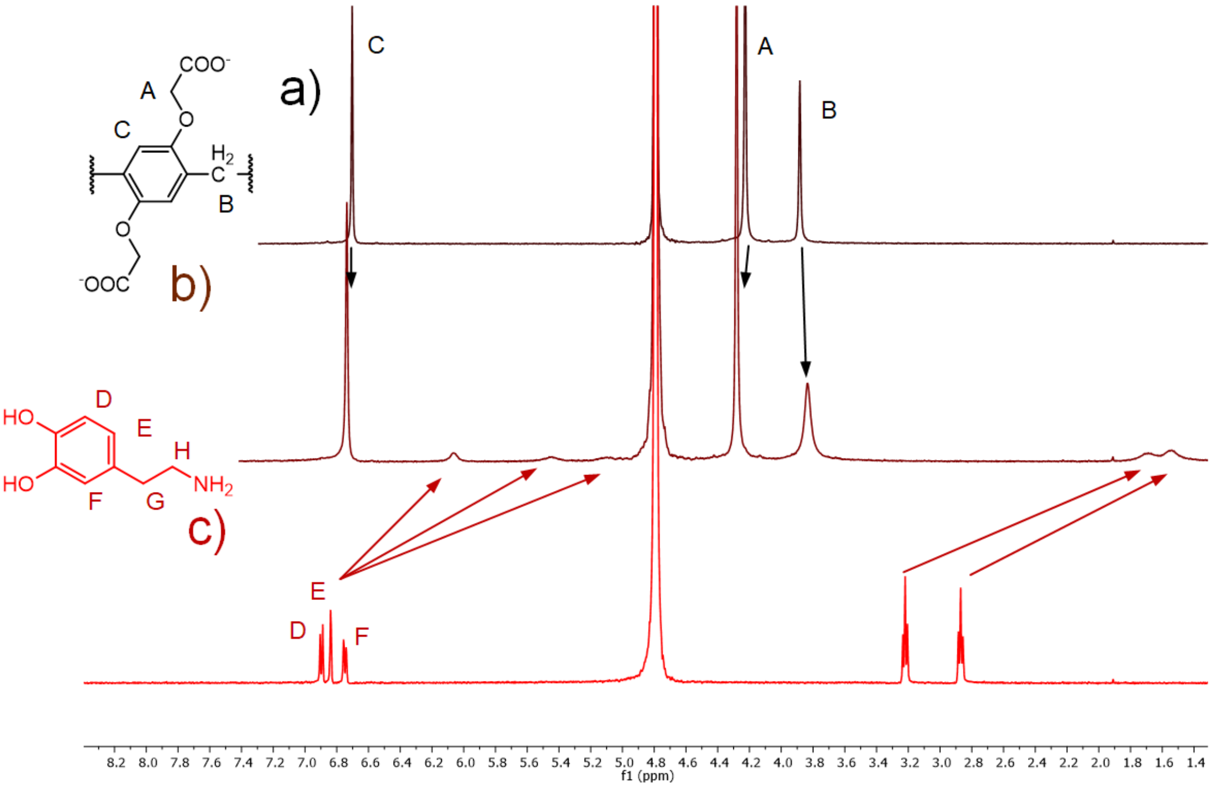

Figure S9. ^{1}H-NMR spectra (500 MHz, D_2O, 1 mM) of a) **WP6** b) **WP6** + dopamine c) dopamine. The dashed ovals show the disappearing signals
